# PPAR in Cardiovascular Disorders

**DOI:** 10.1155/2016/6293629

**Published:** 2016-07-26

**Authors:** Alexander N. Orekhov, Nigora Mukhamedova, Ekaterina A. Ivanova, Manfredi Rizzo

**Affiliations:** ^1^Laboratory of Angiopathology, Institute of General Pathology and Pathophysiology, Russian Academy of Sciences, Moscow 125315, Russia; ^2^Institute for Atherosclerosis Research, Skolkovo Innovation Center, Moscow 121609, Russia; ^3^Laboratory of Lipoproteins and Atherosclerosis, Baker Heart Research and Diabetes Institute, Melbourne, VIC 3004, Australia; ^4^Department of Development and Regeneration, Katholieke Universiteit Leuven (KU Leuven), 3000 Leuven, Belgium; ^5^Unit of Diabetes and Cardiovascular Prevention, School of Medicine, University of Palermo, 90127 Palermo, Italy

Peroxisome proliferation-activated receptors (PPARs) are ligand-inducible transcription factors that, upon binding their ligands, translocate into the nucleus, where they regulate transcription of numerous genes that have the peroxisome proliferator response element (PPRE) in the promoter region [1].

In humans, there are 3 PPAR isoforms: PPAR-*α*, PPAR-*β*/*δ*, and PPAR-*γ*. The isoforms have partially overlapping spectra of activity and are differently expressed in organs and tissues [2]. PPAR-*α* is expressed mostly in tissues characterized by high catabolic activity, including skeletal muscle, liver, proximal tubular cells in kidneys, and brown fat. This PPAR isoform regulates components of *β*-oxidation pathway, enzymes, and transporters involved in fatty acid metabolism and promotes lipolysis and fatty acid oxidation. PPAR-*α* can be activated by fatty acids, prostaglandins, fibric acid derivatives (fibrates), and a number of recently developed specific agonists. Activation of PPAR-*α* has a beneficial effect on processes involved in the development of atherosclerosis, as it decreases plasma triglyceride levels, increases high density lipoprotein cholesterol, and reduces inflammatory response. Therefore, PPAR-*α* agonists gain attention as potential components of antiatherosclerotic therapy [3, 4].

PPAR-*β*/*δ* is expressed in many organs and tissues, with relatively high levels present in skeletal muscle, liver, kidney, and macrophages. It is activated by fatty acids and carbaprostacyclin, stimulates fatty acid oxidation, and improves insulin sensitivity in insulin-resistant animal models. This isoform is also known to have potential antiatherosclerotic properties and is considered for treatment of cardiovascular disorders [5, 6].

PPAR-*γ* is mainly expressed in white and brown fat and can also be found in other organs and tissues, including liver, kidney, and immune cells. It is activated by fatty acids implicated in regulation of glucose homeostasis, lipid metabolism, and adipogenesis. Synthetic PPAR-*γ* agonists, thiazolidinediones, such as pioglitazone and rosiglitazone, are currently used as insulin sensitizers but can have a broader therapeutic potential for treatment of conditions associated with increased cardiovascular risk [7].

Therefore, PPARs have a wide spectrum of biological activities relevant to prevention and treatment of cardiovascular diseases. Moreover, the availability of natural and synthetic small molecule agonists, many of them being relatively well studied by now, makes PPARs attractive therapeutic targets. To date, PubMed literature database delivers more than 3300 articles found by key words “PPAR” + “cardiovascular”. In this special issue, we are happy to present several important works revealing various aspects of PPAR involvement in cardiovascular conditions. The importance of PPAR-*α* signalling for regulation of cardiomyocyte metabolism is highlighted by the research articles of E. Czarnowska with coauthors, who studied the correlation of PPAR-*α* activity and cardiomyocyte function during heart failure, and J. Yang with coauthors, who demonstrated that PPAR-*α* upregulation mediated the effect of testosterone replacement on cardiac metabolic remodelling after myocardial infarction. G. Barreto-Torres with S. Javadov and W.-Y. Wei with coauthors presented the links between PPAR activation and the key cellular signalling network, which includes the AMPK and AKT pathways and regulates cellular metabolism, growth, and response to stress. Another evidence of anti-inflammatory properties of pioglitazone in patients with drug eluting stents is presented by Z. Wang and coauthors. H.-J. Liu and coauthors provide evidence for the critical role of PPAR-*γ* in cardiac fibrosis, and A. Pleskovič with coauthors reported that PPAR-*γ* polymorphisms have a minor effect on atherosclerosis markers in diabetic patients. K.-D. Wagner with coauthors demonstrates that inducible vascular-specific overexpression of PPAR-*β*/*δ* causes cardiac hypertrophy. Finally, a review article of W.-S. Lee and J. Kim provides an overview of the roles of PPARs in the heart. Together, the works collected in this special issue add to our growing knowledge on the PPARs and their activators in the context of cardiovascular disorders.

*Alexander N. Orekhov* *Alexander N. Orekhov* *Nigora Mukhamedova* *Nigora Mukhamedova* *Ekaterina A. Ivanova* *Ekaterina A. Ivanova* *Manfredi Rizzo* *Manfredi Rizzo*
